# Advances in Histone Demethylase KDM3A as a Cancer Therapeutic Target

**DOI:** 10.3390/cancers12051098

**Published:** 2020-04-28

**Authors:** Jung Yoo, Yu Hyun Jeon, Ha Young Cho, Sang Wu Lee, Go Woon Kim, Dong Hoon Lee, So Hee Kwon

**Affiliations:** 1 College of Pharmacy, Yonsei Institute of Pharmaceutical Sciences, Yonsei University, Incheon 21983, Korea; jungy619@yonsei.ac.kr (J.Y.); hyun953@naver.com (Y.H.J.); chipmunkcho@gmail.com (H.Y.C.); tkddn407@naver.com (S.W.L.); goun6997@yonsei.ac.kr (G.W.K.); tci30@naver.com (D.H.L.); 2 Department of Integrated OMICS for Biomedical Science, Yonsei University, Seoul 03722, Korea

**Keywords:** KDM3A, histone H3K9 demethylase, KDM3 inhibitor, anti-cancer agent, epigenetic agent

## Abstract

Lysine-specific histone demethylase 3 (KDM3) subfamily proteins are H3K9me2/me1 histone demethylases that promote gene expression. The KDM3 subfamily primarily consists of four proteins (KDM3A−D). All four proteins contain the catalytic Jumonji C domain (JmjC) at their C-termini, but whether KDM3C has demethylase activity is under debate. In addition, KDM3 proteins contain a zinc-finger domain for DNA binding and an LXXLL motif for interacting with nuclear receptors. Of the KDM3 proteins, KDM3A is especially deregulated or overexpressed in multiple cancers, making it a potential cancer therapeutic target. However, no KDM3A-selective inhibitors have been identified to date because of the lack of structural information. Uncovering the distinct physiological and pathological functions of KDM3A and their structure will give insight into the development of novel selective inhibitors. In this review, we focus on recent studies highlighting the oncogenic functions of KDM3A in cancer. We also discuss existing KDM3A-related inhibitors and review their potential as therapeutic agents for overcoming cancer.

## 1. Introduction

Histone modifications are dynamically controlled by chromatin-modifying enzymes that read specific positions and add or remove corresponding covalent modifications. Among the several different types of enzymes, lysine-specific histone methyltransferases catalyze reversible mono-, di-, and tri-methylation via an S-adenosyl-L-methionine (SAM)-dependent mechanism [1,2]. Conversely, lysine-specific histone demethylases (KDMs) remove these methylation marks [3], contributing to the plasticity of covalent histone modifications. Multiple functional domains are essential for KDM activity. Most KDMs contain a catalytic Jumonji C (JmjC) domain that also functions as a binding pocket for catalytically necessary cofactors [4]. As a result, KDMs are divided into two classes according to the type of cofactor used in their catalytic mechanisms, namely flavin adenine dinucleotide (FAD) [5] or α-ketoglutarate (α-KG also called 2-oxoglutarate (2-OG)) with iron Fe(II) [6]. FAD-dependent enzymes form the KDM1 or Lys-specific demethylase (LSD) subfamily [5], whereas α-KG and Fe(II)-dependent enzymes form JmjC containing demethylase [6,7]. Compared with the restricted catalytic activity of KDM1 at mono- and dimethyl Lys residues, the second class of demethylases is capable of removing all three possible methylation states of methylated Lys residues [8]. Of the 30 known KDMs, 28 of the enzymes belong to the JmjC-KDM class [9], which is divided into five subfamilies: KDM2/7, KDM3, KDM4, KDM5, and KDM6.

**Figure 1:**
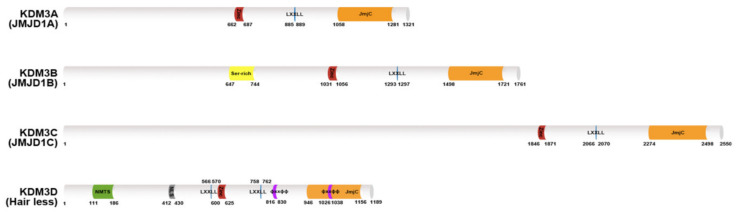
Lysine-specific histone demethylase 3 (KDM3) demethylase protein structures. The KDM3A–D protein architecture consists of a common C_2_HC_4_-type zinc-finger-like motif (red color) LXXLL motif (blue color) and one Jumonji C domain (JmjC) domain (orange color) in the C-terminal catalytic site. KDM3B contains a serine-rich region (yellow color). KDM3D also possesses a nuclear matrix targeting signal (NMTS; green color) a nuclear localization signal (NLS; gray color) and two ϕXXϕϕ motifs (purple color) that serve as thyroid hormone receptor interaction domains.

Human KDM3 is also known as JMJD1 (Jumonji domain-containing 1) and JHDM2 (Jumonji C domain-containing histone demethylase 2). The KDM3 subfamily encompasses four members (Figure 1): KDM3A, KDM3B, KDM3C, and KDM3D. Of these proteins, the enzymatic activity of KDM3C remains controversial [9,10]. KDM3A and KMD3B catalyze the demethylation of transcriptionally repressive mono- and di-methylated histone H3 lysine 9 (H3K9me1/me2) in vitro and in vivo with a preference for dimethylated residues, thereby mediating transcriptional activation [10,11]. KDM3D/hairless (HR) protein demethylates H3K9me1 in vitro and in vivo, and it can also weakly demethylate H3K9me2 in vivo [12]. The KDM3 proteins share a common domain architecture consisting of a JmjC domain at the C-terminus, a catalytic histone demethylase domain [13] and non-catalytic C_2_HC_2_ zinc (Zn) finger, and LXXLL (where L is leucine and X is any amino acid) motifs for interacting with nuclear hormone receptors (NRs). The Zn-finger domain of KDM3A is involved in determining substrate specificity [10,11] but its exact mechanism is unknown. Additionally, the JmjC and the Zn-finger domains are required for forming a homodimer of KDM3A, which is important for its functions [11,14]. Further studies are needed to determine whether other KDM3 proteins undergo homodimerization.

**Figure 2:**
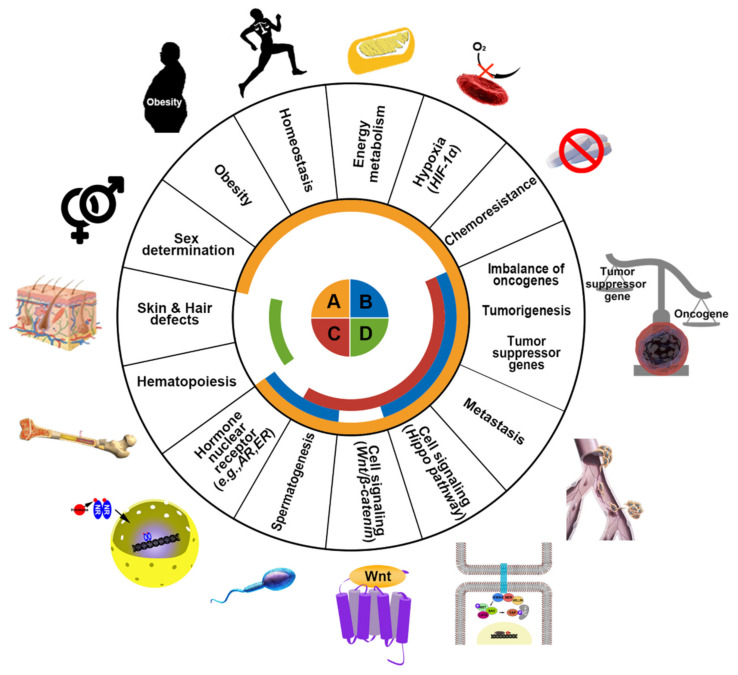
Schematic representation of the major functions of KDM3 in humans. The KDM3 family consists of four proteins: KDM3A (orange) KDM3B (blue) KDM3C (red) and KDM3D (green). These proteins regulate gene expression and chromatin dynamics by demethylating H3K9me1/2. KDM3 family proteins normally function in energy metabolism, homeostasis, obesity, sex determination, hematopoiesis, hormone nuclear receptor signaling, spermatogenesis, and cell signaling pathways. However, dysregulation in KDM3 expression or function promotes skin and hair defects and tumorigenic processes such as cell survival under hypoxia, malfunction of tumor suppressor genes, induction of oncogenes, development of chemoresistance, and metastasis in cancer patients.

KDM3 proteins are deregulated in skin, hair, and cardiovascular diseases and multiple cancers, including breast, prostate, and colon cancers, and lymphomas, and thus, they have emerged as potential broad-spectrum therapeutic targets. Each of these KDM3 proteins plays differential yet redundant functions in both physiological and pathological processes (Figure 2). Among them, KDM3A was first cloned and characterized as a mouse male germ cell-specific transcript in 1991 [15]. KDM3A demethylates H3K9me1/me2 [11] to increase gene transcription and plays key roles in spermatogenesis [16], energy metabolism [17], stem cell regulation [18], and sex determination [19]. Moreover, increasing evidence has indicated the key roles of KDM3A in promoting cancer progression. (1) KDM3A is frequently upregulated in multiple cancers and elevated KDM3A levels are associated with worse cancer prognosis [20,21]. (2) Depletion of KDM3A significantly inhibits colony formation [22,23], cell proliferation [24,25] and xenograft tumor formation [26,27]. (3) The depletion of KDM3A substantially suppresses migration, invasion, and metastasis [28,29]. (4) KDM3A regulates oncogene expression by binding NRs (e.g., AR [20,30], ER [31,32,33], HIF-1α [24,25]) and modulating downstream signaling (e.g., Wnt/β-catenin signaling [26,34] and Hippo signaling [27]).

This review concentrates on recent studies on the potential oncogenic functions of KDM3A subfamily enzymes in cancer. Although there are no reported compounds that are selective for KDM3 subfamily proteins or their isoforms, we have summarized current knowledge and the potential of KDM3 inhibitors as therapeutic agents for cancer treatment.

## 2. Altered Expression and Functions of KDM3A in Cancer

Numerous studies demonstrated that abnormal KDM3 expression promotes the progression of cancer, as substantiated by the number of published studies shown in Table 1. KDM3A and KDM3B are well known to be tightly related to cancer, whereas KDM3C and KDM4D are relatively unstudied in cancer development. Among the four KDMs, this review focuses on the relatively well-studied association between KDM3A and various cancers.

### 2.1. Prostate Cancer (PCa)

KDM3A is overexpressed in PCa [20,30,35]. In prostate adenocarcinoma, androgen-dependent signaling plays a key role in tumorigenesis [20,30]. Ligand bound androgen receptor (AR) translocates into the nucleus upon dimerization, recruiting coactivators to androgen response elements (AREs) to regulate gene expression. As an AR coactivator, KDM3A is significantly enriched in AREs, and it alters the expression of androgen response genes through demethylation [20,30]. Using gene set enrichment analysis, it was identified that overexpressed KDM3A posttranscriptionally activates androgen signaling and cancer metabolic pathways, such as hypoxia and glycolytic pathways, thus upregulating oncogene expression. Moreover, in an orthotopic prostate tumor model using CWR22Rv1 cells with permanently activated AR, KDM3A knockdown disrupts tumor formation [30], highlighting the importance of KDM3A in androgen signaling and carcinogenesis.

In addition to its role in the androgen signaling pathway, KDM3A also regulates alternative splicing of AR pre-mRNA, generating AR variant-7 (AR-V7) [36]. AR-V7 is one of the major mechanisms through which patients with PCa develop resistance to androgen deprivation therapy, which blocks AR activity. However, in patients with castration-resistant PCa (CRPC), AR activity is restored through alternative splicing. KDM3A facilitates the recruitment of spliceosome components and binds to heterogeneous nuclear ribonucleoprotein F, a splicing regulator, to create constitutively active AR-V7. Knockdown of KDM3A in Rv1 and LN95 cells resulted in reduced protein and AR-V7 mRNA levels. In fact, tissue microarray demonstrates that KDM3A promotes AR-V7 expression in certain human PCa tissues. In the KDM3A-knockdown Rv1 xenograft prostate tumor model, tumor weight is decreased by 13-fold, whereas no tumor formation occurs in castrated mice.

KDM3A interacts with and regulates the transcription of both AR and hypoxia-inducible factor 1α (HIF-1α) [24,25]. KDM3A serves as a transcriptional coactivator of HIF1α and AR in the context of hypoxia. Hypoxia increases the occupancy of HIF-1α and KMD3A on the gene encoding prostate-specific antigen (PSA), a secreted chymotrypsin-like serine protease A that cleaves proteins that function in cell proliferation and metastasis. Chromatin immunoprecipitation (ChIP) assay results confirmed that HIF-1α, together with AR, recruits endogenous KDM3A to PSA enhancers. Furthermore, the occurrence of H3K9me1 and H3K9me2, both of which are substrates of KDM3A, on PSA enhancers is reduced under hypoxia, further supporting the idea that KMD3A promotes PCa progression through the epigenetic regulation of PSA by interacting with HIF-1α.

In addition to AR and HIF-1α, c-Myc is posttranscriptionally controlled by KDM3A, leading to cancer proliferation and survival [20,21]. Overexpression of c-Myc is associated with poor prognosis and recurrence of PCa. Knockdown of KDM3A significantly inhibits c-Myc and AR activities. Conversely, double depletion of KDM3A and AR fails to reduce c-Myc levels, whereas KDM3A knockdown without changes of AR levels successfully decreases c-Myc mRNA transcription [21]. KDM3A also regulates HUWE1, an E3 ubiquitin ligase known to target Myc increases the stability of c-Myc [20]. KDM3A inhibits HUWE1/c-Myc interaction and prevents the HUWE1-dependent degradation of c-Myc, thus increasing c-Myc levels and PCa cell proliferation. Furthermore, c-Myc knockdown phenocopies the effects of KDM3A knockdown in PCa cells, whereas c-Myc overexpression in KDM3A-depleted cells partially regain the ability to grow and proliferate both in vitro and in vivo. Furthermore, the expression levels of c-Myc are positively correlated with those of KDM3A in certain PCa subset specimens. KDM3A depletion strongly inhibits PCa cell growth, so KDM3A can serve as a potential target in the development of novel drugs for treating PCa.

### 2.2. Breast Cancer (BCa)

KDM3A is overexpressed in BCa tissues. The Cancer Genome Atlas (TCGA) human BCa database (*n* = 729) shows that patients with BCa and high KDM3A expression have a three-fold higher risk of death than those with normal or low KDM3A expression [37]. Two-thirds of patients with BCa express ER and require ER-mediated transcriptional activation for cancer survival [38]. Similar to KDM4B, KDM3A is a well-known key ER coregulatory protein in ER signaling [31,32,33]. KDM3A acts as a positive regulator of ER, and its absence impairs ER recruitment to the cis-regulatory elements of the target gene promoters. Global gene expression analysis demonstrated that KDM3A regulates approximately 42% of ER target genes [32]. KDM3A depletion using siRNA notably downregulates ER target genes GREB1 and pS2 regardless of the presence of the ER ligand, β-estradiol (E2). The absence of KDM3A elevates H3K9me2 levels at both GREB1 and pS2 estrogen response elements (EREs) even in the presence of E2, failing to remove the repressive marks of these loci. Additionally, KDM3A knockdown abrogates the recruitment of ER to enhancer regions of ER target genes such as *CCND1 (Cyclin D1)*, *MYC*, and *XBP1*, thereby highlighting the importance of KDM3A in ER signaling [32,39]. Likewise, in Kdm3a knockout mice, CCND1 mRNA expression is decreased in Kdm3a knockout mouse mammary gland tumors, compared with that in wild-type mice [40]. Furthermore, BCa cells expressing catalytically inactive KDM3A exhibit downregulation of cell cycle regulatory genes and increased proliferation, confirming the role of KDM3A in BCa progression [32].

In addition to its single role as a coactivator, KDM3A also cooperates with KDM4B via an auto-regulatory loop to regulate the functions of ER by facilitating the recruitment of other ER coactivating proteins and ER transcriptional complexes to the EREs [33]. Both KDM3A and KDM4B are enriched at EREs of ER target genes, such as *pS2*, *GREB1*, and *CCND1*. Notably, the chromatin occupancy of KDM3A is depleted by the absence of KDM4B and vice versa, strongly suggesting that these demethylases need each other to facilitate their association with chromatin. However, the mRNA and protein levels of KDM3A and KDM4B exhibit different patterns. Although depletion of KDM4B downregulates KDM3A mRNA and protein levels in certain BCa cells, KDM4B protein expression is not affected by KDM3B knockdown in BCa cells, suggesting a regulatory mechanism in which KDM4B regulates KDM3A expression. In addition to KDM4B, forkhead box protein A1 (FOXA1) is closely related to KDM3A in BCa. FOXA regulates chromatin binding and activation of ER target genes. Through ChIP assays, it has been determined that promoter and enhancer enrichment of FOXA1 on ER target genes *pS2* and *GREB1* are reduced upon KDM3A knockdown using siRNA in BCa cells. Because the chromatin binding of KDM3A precedes ER recruitment, Jones et al. speculated that KDM3A acts to deposit FOXA1 at EREs by demethylating H3K9me1/2, which in turn would bring KDM4B to the ER target genes and make up the transcriptional complex. Additionally, microarray analysis indicated that KDM3A, KDM4B, and FOXA1 have overlapping gene regulatory profiles for proliferative genes in BCa cells. Thus, KDM3A facilitates KDM4B recruitment to EREs, whereas KDM4B regulates KDM3A and FOXA1 expression, resulting in an overlap of the transcriptomes of these three proteins.

KDM3A is responsible for the chemoresistance and stemness in BCa by removing Lys372me1 on p53 and H3K9me2 [28] and controlling homeobox protein Hox-A1 (HOXA1) expression [31]. Methylation of p53 at Lys372 by SET9 histone lysine N-methyltransferase activates p53, causing it to enter and localize in the nucleus, in which it activates its target genes [28]. KDM3A inhibits such activity of p53 by demethylating Lys372, thus hampering p53-mediated pro-apoptosis and resistance to paclitaxel and cisplatin. Knockdown of KDM3A increases p53K372me1 levels in BCa cells, and Co-immunoprecipitation (Co-IP) analysis illustrated that KDM3A interacts with p53. Consistent with these results, knockdown of p53 in KDM3A knockdown cells abolished apoptotic gene expression, including *CDKN1A (p21)*, *BAX*, *PUMA*, and *NOXA*, and re-established paclitaxel sensitivity. In addition to paclitaxel, tamoxifen resistance is caused by KDM3A regulated HOXA1 [31]. KDM3A is directly associated with activated CDC42 kinase 1 (ACK1), a non-receptor tyrosine kinase that functions in tumorigenesis, resistance, and metastasis and acts as an integrator of various signaling pathways such as HER2 signaling. ACK1 and KDM3A form a complex upon coexpression and ACK1 phosphorylates KDM3A at Tyrosine 1114 in BCa cells. Phosphorylated KDM3A regulates HOXA1, a potent oncogene that induces the formation of aggressive, immortal mammary epithelial cells, by demethylating HOXA1 promoter and activating its transcription. As a result, targeting KDM3A in patients with BCa can be a powerful strategy to overcome drug resistance and therefore cancer recurrence.

Despite the numerous aforementioned studies on KDM3A’s association with BCa survival and poor prognosis, one study by Yao states that KDM3A expression does not correlate with malignant characteristics of BCa in clinicopathological parameters [41]. Patterns of KDM3A staining in BCa tissues failed to show significant association with clinical stage, pathological grade, tumor size, and the expression of ER and progesterone receptor (PR) status. Furthermore, BCa patients with high KDM3A expression did not exhibit a meaningful association in BCa prognosis when compared to those with low KDM3A expression. However, this study failed to pair normal control tissues with the corresponding BCa tissues.

Nonetheless, most, if not all, studies demonstrated that KDM3A is associated with the progression of BCa metastasis [28,29,32] by removing methylation marks on pro-invasive genes. KDM3A promotes the metastasis of BCa by interacting with a Brahma related gene 1 (BRG1), a mammalian chromatin remodeling complex, on the Mucin 1 (MUC1) promoter to remove repressive methylation marks and activate pro-invasive gene transcription [29]. BRG1 contributes to BCa metastasis via tumor necrosis factor-α (TNF-α) and interferon-gamma (IFN-γ) induced transcription of *MUC1*, which is a pro-metastatic oncoprotein that is aberrantly elevated in highly malignant BCa due to transcriptional upregulation. Such upregulation of MUC1 is attributed to the interaction of KDM3A with and recruitment by BRG1 on the MUC1 promoter in a signal transducer and activator of transcription 1 (STAT1)- and p65-dependent manner. Coexpression of BRG1 and KDM3A additively activates MUC1 transcription and decreases H3K9me2 levels on the MUC1 promoter, causing inflammation-induced migration and invasion of BCa cells. In support, a ChIP assay revealed that KDM3A is enriched on the promoter of matrix metalloprotease 9 (MMP-9), a gene related to cell motility and migration, and that its H3K9me2 levels are decreased in the presence of KDM3A [28]. Moreover, the target genes of activator protein 1 (AP-1) transcription factor, which regulates cell migration proteins MMP-9 and S100A, are not expressed when KDM3A is absent, confirming the role of KDM3A in BCa invasion and metastasis.

### 2.3. Colon Cancer

Similar to the abnormally elevated expression of KDM3 in PCa, KDM3A is also overexpressed in colon cancer, and its levels are increased in colorectal cancer (CRC) metastatic lesions [26,34,42,43]. According to TCGA data, KDM3A is significantly overexpressed in colon adenocarcinomas, and its expression is correlated with reduced survival rates and high rates of recurrence. KDM3A is present in both the cytoplasm and nucleus in CRC and head and neck cancers, but it is rarely detected in the nuclear matrix and heterochromatin regions [42,44]. Downregulation of KDM3A in HCT116 CRC cells inhibits the clonogenic activity of CRC cells [42,45], arrests cell cycle progression, suppresses CRC cell proliferation and migration, and reduces xenograft tumor formation [26,27]. Moreover, as identified through transcriptome analysis, KDM3A downregulation stimulates p53- and transforming growth factor-β1 (TGF-β1)-related tumor suppressor pathways and inhibits c-Myc- and N-myc-driven oncogenic pathways.

Hyper-activated Wnt/β-catenin signaling is associated with CRC recurrence, development of drug resistance [26,34] and metastasis [27,43]. Among KDM3 family proteins, KDM3A and KDM3B play crucial roles in activating the Wnt signaling pathway [34]. The absence of KDM3A or KDM3B inhibits the Wnt target genes *AXIN2* and *DKK1*. Moreover, both KDM3A and KDM3B directly interact and recruit β-catenin [26] in HCT116 CRC cells and specifically remove H3K9me2 from the Wnt target gene promoters, controlling the tumorigenic ability of human colorectal CSCs [26,34]. KDM3A knockdown downregulates both the protein and mRNA levels of c-Myc, cyclin D1, and MMP-9, which are all downstream targets of the Wnt/β-catenin signaling pathway [26]. Abnormal regulation of c-Myc and MMP-9 promotes cancer cell proliferation and metastasis, respectively. Furthermore, the expression levels of KDM3A positively correlate with β-catenin target genes in CRC specimens from TCGA data and predictive of worse cancer outcomes.

Recently, it was found that the oncogenic role of KDM3A is regulated by acetylation on enhancers of Hippo target genes [46] and PHF5A [27]. KDM3A recruits p300 to Hippo target gene enhancers and facilitates the interaction between TEA domain transcription factor 1 (TEAD1), a Hippo pathway transcription factor, and yes-associated protein 1 (YAP1) by demethylating H3K9me1/2 on YAP1 enhancers [46]. Expression of Hippo target genes such as *WEE1*, *IFRD1*, *RND3*, *PARD6B*, *ELMSAN1*, *UGDHAS1* correlates with KDM3A expression in CRC patients, leading to tumorigenesis. As activated in the Hippo pathway, upon starvation, PHF5A, a component of U2 small nuclear ribonucleoproteins (snRNPs) of the spliceosome, is acetylated at Lys29 by p300 [27]. This acetylation enhances the interaction of PHF5A with U2 snRNPs and stabilizes KDM3A mRNA by reducing the abnormal retention of KDM3A intron 3, which carries a stop codon. Moreover, both KDM3A and c-Myc protein expression levels are significantly upregulated in PHF5A-K29Q HCT116 cells, indicating that PHF5A Lys29 acetylation decreases CRC tumorigenesis by regulating the alternative splicing of KDM3A. Notably, PHF5A Lys29 acetylation and KDM3A upregulation are correlated with the poor prognosis of CRC. Thus, PHF5A acetylation induces the stabilization of KMD3A mRNA and enhances its expression, in which the protein consequently activates signaling pathways that promote CRC.

In addition to its role in activating Wnt/β-catenin and Hippo signaling pathways in colon cancers, KDM3A activates the gene transcription of 15-lipoxygenase-1 (15-LOX-1), which is silenced in cancer cells [47]. 15-LOX-1 regulates terminal cell differentiation and inflammation by producing multiple inflammation-regulatory lipid signaling mediators. In CRC cells, 15-LOX-1 is abnormally downregulated, but re-expression of 15-LOX-1 inhibits cell growth and restores its function in cell differentiation and apoptosis. KDM3A is recruited to the 15-LOX-1 promoter and is required for its transcription activation. siKDM3A treated CRC cells exhibit reduced KDM3A interactions and 15-LOX-1 mRNA levels. Accordingly, H3K9me2 levels on the 15-LOX-1 promoter are increased, even in the presence of depsipeptide, which facilitates the recruitment of KDM3A on 15-LOX-1 promoters in CRC cells. Overall, these findings support the indispensable function of KDM3A in colonic tumorigenesis, thus illuminating its potential as a novel therapeutic target for inhibiting cancer growth.

### 2.4. Lung Cancer

KDM3A is suggested to aid lung adenocarcinoma cells in evading the immune system of patients by upregulating forkhead box P3 (Foxp3) expression in regulatory T cells (Tregs) [48]. Foxp3 expression, controlled by activated Toll-like receptor 4 (TLR4), enhances immunosuppressive functions of Tregs by inducing inhibitory cytokine secretion and facilitate lung cancer cells to escape the immune system. Upregulation of TLR4 significantly increases the expression of KDM3A in A549 lung adenocarcinoma cells, which subsequently controls Foxp3 transcription. Knockdown of KDM3A in normally active TLR4 lung adenocarcinoma cells reduces Foxp3 expression and downstream cytokines such as TGF-β1 and interleukin-35 (IL-35), as well as heme oxygenase 1. Furthermore, the colocalization of KDM3A and Foxp3 and suppressed luciferase activity under the control of Foxp3 promoters upon KDM3A depletion further support that KDM3A regulates Foxp3 transcription by directly binding to its promoter. Despite these results that support the role of KDM3A in evading the immune system, further studies are needed to fully clarify its role in promoting lung adenocarcinoma growth.

As noted in breast cancers [29], KDM3A in lung cancer is also recruited to proliferation- and metastasis-related gene promoters by BRG1 [49]. BRG1 is pivotal for the expression of cyclin B1 and latent TGF binding protein 2 (LTBP2), the upregulation of which is correlated with malignant lung cancer growth. In lung cancer, hypoxia induces BRG1 to regulate the proliferation and migration of lung cancer cells by activating cyclin B1 and LTBP2, whereas loss of BRG1 via siRNA treatment decreases hypoxia-induced migration and proliferation. A series of ChIP assays indicated that hypoxia increases the interaction with and recruitment of KDM3A by BRG1 to proximal cyclin B1 and LTBP2 promoters. KDM3A recruitment to these promoters is decreased when BRG1 expression is abrogated, confirming that KDM3A interacts with BRG1 and controls the methylation status on these genes. Taken together, these data highlight the common role of KDM3A in different cancers, further reinforcing its prominent role in tumorigenesis.

### 2.5. Liver Cancer

The level of KDM3A in hepatocellular carcinoma (HCC) is higher than that in normal tissues (n = 110) [50]. HCC samples with high KDM3A expression have a higher recurrence rate than those with low expression (n = 47). The expression level of KDM3A is an independent predictor of recurrence but is not associated with any clinicopathological features. Knockdown of KDM3A inhibits HCC cell growth, invasion, and epithelial-mesenchymal transition under hypoxia. Furthermore, KDM3A upregulation promotes hypoxia-induced HCC proliferation and increases the expression of its target gene *adrenomedulin* by decreasing H3K9me2 levels at the target promoter [51]. The depletion of KDM3A by shRNA treatment suppresses the growth of HCC HepG2 tumor xenografts in vivo. In addition to tumorigenesis, KDM3A is involved in radioresistance in HCC cells. Downregulation of KDM3A and KDM4B by treatment with the plant-derived polyphenol emodin (1, 3, 8-trihydroxy-6-methylanthraquinone) reduces radioresistance in HCC cells under both normoxia and hypoxia [52]. Collectively, these results suggest that KDM3A increases the malignant potential of HCC and represents an applicable prognostic marker.

Furthermore, KDM3A is linked to oncogenic pathways in liver cancer. KDM3A contributes to hepatotumorigenesis through the phosphatidylinositol 3-kinase (PI3K) pathway, which is 30%–50% activated in HCC [53]. Knockout of Kdm3a inhibits tumor formation in *Pik3ca* transgenic mouse livers [54]. Loss of Kdm3a decreases the PI3K-activated expression of AP-1 target genes. Kdm3a promotes the recruitment of c-Jun to the AP-1-binding sites of *Cd44*, *Mmp7*, and *Pdgfrb* target genes without altering c-Jun expression. In addition, the interaction between Kdm3a and c-Jun facilitates the binding of BRG1, a component of the SWI/SNF chromatin remodeling complex, to AP-1 transcription sites. Furthermore, coexpression of KDM3A and c-JUN is observed in human premalignant lesions with PI3K activation. Notably, Kdm3a is highly expressed in Cd44 (a liver CSC marker)-positive hepatocytes, and it controls the number and tumor-initiating potential of Cd44-positive cells. Thus, these data suggest that KDM3A plays a critical role in liver cancer initiation and may be an attractive therapeutic target for a subset of HCCs related to activated PI3K signaling.

### 2.6. Bladder Cancer

KDM3A expression is significantly higher in human bladder cancer cell lines and tissues than in nontumor bladder tissues [22,55]. KDM3A overexpression activates HOXA1 transcription followed by that of CCND1, promoting cell proliferation and survival through the G1/S transition. KDM3A protein actively binds to the promoter of HOXA1 and demethylates H3K9, increasing H3K9me2 levels but not H3K9me3 levels. Interestingly, the level of KDM3A is relatively similar in different stages and grades of cancer. Such consistency indicates that KDM3A overexpression starts in the early stage and remains high throughout cancer progression, suggesting that KDM3A is an essential contributor to cancer development, particularly even under normoxic conditions. Downregulation of KDM3A and CCND1 by KDM3A siRNA inhibits cell growth and proliferation by cell cycle arrest. Strikingly, KDM3A and HOXA1 exhibit strong correlations in bladder cancer tissues (*n* = 55) and cell lines (*n* = 18), further supporting that KDM3A contributes to bladder cancer progression by regulating HOXA1.

KDM3A cooperates with HIF-1α, the major transcription factor of genes involved in glucose metabolism [55]. Knockdown of KDM3A hinders glycolysis by reducing the expression of genes related to glucose metabolism such as *GLUT1*, *HK2*, *LDHA*, *MCT4*, *PGM*, and *PGK1*. For instance, depletion of KDM3A decreases the expression of PGK1 by inhibiting H3K9me2 demethylation at the promoter. When KDM3A loses its demethylase activity via knockdown or mutation, it no longer cooperates with HIF-1α, and the increment of gene expression essential for glycolysis is not observed. Conversely, overexpression of KDM3A increases the expression of various important glycolytic genes and the glycolysis rate. Moreover, KDM3A and several major glycolytic genes mentioned above display a positive correlation in bladder cancer specimens from GEO profiles and TCGA datasets. On the other hand, KDM3A downregulation decreases bladder cancer proliferation, in vitro colony formation, and in vivo xenograft tumor growth. As a result, KDM3A promotes bladder cancer progression by enhancing glycolysis via coactivating HIF-1α and may serve as a potential target for bladder cancer treatment.

### 2.7. Neuroblastoma

KDM3A is upregulated in neuroblastoma [56], which is the most common pediatric extracranial tumor originating from precursor neuroblast cells [57]. The *MYCN* oncogene is amplified and overexpressed in neuroblastoma, and overexpression of N-myc was observed in one-fourth of patients with neuroblastoma. Tee et al. revealed that N-myc upregulates KDM3A expression levels in N-myc-amplified neuroblastoma cells. N-myc functions at the KDM3A core promoter by directly binding to a canonical Myc-response element enhancer box (E-box). N-myc indirectly modulates the expressions of other genes in part by upregulating KDM3A expression. KDM3A, but not N-myc, binds to the long non-coding RNA (lncRNA) metastasis associated lung adenocarcinoma transcript 1 (MALAT1) core promoter and upregulates its expression by demethylating H3K9me2. Additionally, KDM3A and MALAT1 promote the migration and invasion of neuroblastoma cells, whereas the small-molecule pan-KDM inhibitor dimethyl N-oxalylglycine (DMOG) (2-OG cofactor inhibitor) inhibits such effects. As a result, N-myc induces neuroblastoma cell migration and invasion by modulating KDM3A and MALAT1 levels, providing the evidence for the development of a KDM3A-selective inhibitor to block neuroblastoma metastasis.

### 2.8. Pancreatic Cancer

KDM3A is overexpressed in pancreatic tumor cell lines and tissues compared with that in adjacent non-tumor tissues such as islet and acinar cells [23]. KDM3A knockdown in pancreatic cancer cells considerably reduces colony formation, spheroid formation, migration, and invasion compared with the findings in control cells. In addition, in mice, downregulation of KDM3A inhibits the growth of orthotopic tumors. Conversely, KDM3A overexpression induces the formation of pancospheres in human non-cancerous pancreatic ductal cells and tumor formation and causes metastasis in mice. KDM3A upregulates doublecortin calmodulin-like kinase 1 (DCLK1), a marker of pancreatic CSCs, by binding to the promoter and removing H3K9me1. Consequently, knockdown of KDM3A expression decreases DCLK1 levels. KDM3A is increased under hypoxia through pancosphere formation in pancreatic cancer cells, thereby increasing the DCLK1 mRNA level. Both KDM3A and DCLK1 mRNA levels are higher in human pancreatic tumor tissues than in non-tumor pancreatic tissues, and their expression is correlated with shorter survival times of patients with pancreatic cancer. These findings indicate that KDM3A plays a pivotal role in pancreatic cancer cell progression and stem cell properties and targeting KDM3A represents a promising novel therapeutic strategy for pancreatic cancer.

### 2.9. Ovarian Cancer

Upregulation of KDM3A in platinum-resistant ovarian cancer contributes to ovarian cancer stemness and chemoresistance [58]. High expression of KDM3A facilitates ovarian cancer growth and survival, as its depletion leads to G2/M cell cycle arrest, senescence, and apoptosis. In addition, KDM3A demethylates p53K372me1 and stabilizes the binding of p53 to the promoters of p21 and B-cell lymphoma 2, which are both pro-apoptotic genes. Furthermore, KDM3A contributes to ovarian cancer stemness in cisplatin-resistant cells through the regulation of pluripotent proteins such as Sox2, Nanog, Oct4, and Lin28. KDM3A knockdown in cisplatin-resistant cells reduces Sox2 and Nanog expressions but increases H3K9me2 levels in Sox2 promoters. Therefore, KDM3A epigenetically activates Sox2 expression and promotes ovarian cancer stemness by removing repressive histone marks. In addition to in vitro results, KDM3A depletion also significantly hampers ovarian cancer growth in vivo. In a tumor xenograft mouse model, both the tumor growth and volume were diminished in KDM3A-depleted tumors. Moreover, KDM3A and Sox2 expression are both increased in ovarian cancer than in the normal tissues, and their expression is positively correlated with each other. These findings suggest that KDM3A serves as a critical mediator of chemoresistance and CSC growth in ovarian cancer, through posttranslational regulation of p53, pro-apoptotic genes, and pluripotency genes. Therefore, small molecules or drugs targeting KDM3A represent potential therapeutics for treating chemoresistant ovarian cancer.

### 2.10. Multiple Myeloma (MM)

KDM3A expression is upregulated in MM cell lines and patient samples compared with the findings in normal plasma cells [59]. Knockdown of KDM3A notably inhibits MM cell growth in vitro and in vivo and induces MM cells’ apoptosis. KDM3A directly upregulates the expression of krüppel-like factor 2 (KLF2) and interferon regulatory factor 4(IRF4), which are essential transcription factors for MM cell survival, by removing H3K9me1/me2 from their promoters. Moreover, although KLF2 and IRF4 form a positive feedback loop and reciprocally transactivate each other, overexpression of either KLF2 or IRF4 does not reverse cell growth inhibition mediated by KDM3A depletion. Notably, knockdown of KDM3A, IRF4, or KLF2 reduces both MM cell adhesion and MM cell homing to bone marrow stromal cells and bone marrow, respectively, by decreasing integrin beta 7 (ITGB7) expression. In sum, these results suggest that the KDM3A-KLF2-IRF4 pathway critically contributes to MM cell survival and homing to the bone marrow, making this axis a potentially valuable therapeutic target for MM.

Hypoxia-inducible KDM3A [60] is most significantly upregulated by HIF-1α accumulation in MM cells [61]. Knockdown of KDM3A induces apoptosis in MM cells in chronic hypoxia. Recently, Ikeda et al. identified MALAT1 as a KDM3A target gene, and this lncRNA plays a critical oncogenic role in many cancer types [62]. As observed in gastric cancer [56], KDM3A, but not HIF-1α, promotes MALAT1 expression by demethylating H3K9 at its promoter under hypoxic conditions. Upon hypoxia, increased MALAT1 expression induces the acquisition of an anti-apoptotic phenotype, leading to increased expression of glycolytic genes such as *PFKFB3*, *PFKFB4*, and *SLC2A1* independently of IRF4. These results indicate that hypoxia-inducible KDM3A enhances the expression of glycolytic genes by controlling MALAT1 in MM. Together, these findings indicate that the HIF-1α-KDM3A-MALAT1 has a critical role in the acquisition of the antiapoptotic phenotype of MM.

## 3. KDM3 Inhibitors as Emerging Epigenetic Cancer Agents

**Figure 3:**
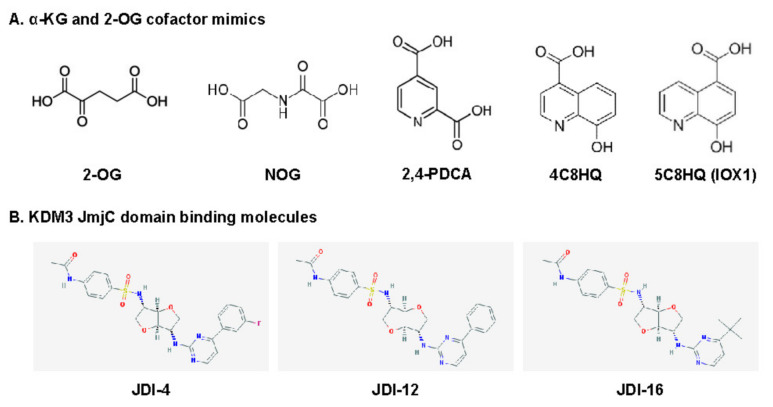
Chemical structures of KDM3 inhibitors and modulators: **(a)** cofactor mimics; **(b)** KDM3 JmjC domain-binding molecules.

Deregulation of KDM3 catalytic activity and expression is increasingly associated with the progression of various cancers. Indeed, extensive histone methylation modifications imply chemoresistance [28,58], patient relapse [20,21,26,31,34,50], and poor prognosis [20,21,46]. KDM3 proteins, particularly KDM3A, can act as oncoproteins, making them potential therapeutic targets for cancer treatment. KDM inhibitors can be categorized into four classes based on their catalytic mechanisms [63]; α-KG/2-OG cofactor mimics, metal cofactor disruptors, competitive substrate inhibitors, and substrate- and cofactor-independent inhibitors. The development of potent selective inhibitors for KDM is going through an active investigation, but only a few KDM3 inhibitors have been reported and they are pan-KDM inhibitors that are not selective for KDM3 subfamily. Recently, a few KDM3 modulators were reported as KDM3 JmjC domain-binding molecules (Figure 3).

### 3.1. α-KG/2-OG Cofactor Mimics: Metal-Chelated Inhibitors

α-KG/2-OG is required for the demethylation of all JmjC-KDMs [4,6,8]. Most KDM inhibitors are of the first class that includes broad-spectrum α-KG/2-OG cofactor inhibitors. These inhibitors competitively bind Fe(II) (ferrous iron) molecules at the active catalytic site and inhibit all major regions of the JmjC-KDM proteins through the enzymes’ conserved Fe(II) and α-KG/2-OG sites [64,65]. Most α-KG/2-OG cofactor inhibitors are α-KG/2-OG competitors, including the oxayly acid-derivative N-oxalylglycine (NOG) [66], 2,4-pyridinedicarboxylates (2,4-PDCA) [37,67,68,69], hydroxamate [67], and hydroxyquinoline (HQ) derivatives [70] (Figure 3A). NOG was first identified as a 2-OG oxygenase inhibitor, and it is a closely related analog of α-KG/2-OG [71]. Hopkinson et al. investigated the comparative inhibition profiles for NOG, 2,4-PDCA, 5-carboxy-8-hydroxyquinoline (IOX1), and 4-carboxy-8-hydroxyquinoline (4C8HQ) for 14 active 2-OG oxygenases, including KDM3A [70], by in vitro assays. All four of the tested compounds inhibited every 2-OG oxygenases. However, the degree of inhibition for KDM3A varies substantially across the four inhibitors. Among the four inhibitors tested, IOX1 was the most potent inhibitor as it displayed IC_50_ values at 0.2 μM or lower against the KDM3A. IOX1 causes iron translocation in protein’s active-site metal position with respect to the position when the inhibitor is not present. This iron translocation may contribute to the variance in the efficiency of IOX1 and 4C8HQ, a regioisomer of IOX1 [72]. Although α-KG/2-OG cofactor analogs have proven to be broad-spectrum inhibitors, these compounds apparently lack the selectivity for each 2-OG oxygenase, including KDM and have poor cell penetration because of their structures.

### 3.2. Metal Cofactor Disruptor

KDM’s catalytic activity can be inhibited by interfering with iron cofactor binding using metal cofactor disruptors such as non-iron metals and organic molecules. Non-iron metals including nickel inhibit the catalytic activity of KDM3A by replacing the Fe(II) ion in the iron-binding pocket at the catalytic site to block the hydroxylation process [73]. The IC_50_ of nickel ions for KDM3A is 25 μM. However, many proteins contain nickel ion-binding sites and thus, the selectivity of iron-ejecting compounds for KDM3A requires further study.

### 3.3. KDM3 Modulators: KDM3 JmjC Domain-Binding Molecules

Recently, Xu et al. reported new KDM3 modulators from a library of 149,519 natural products and 33,765 Chinese medicine components via virtual screening using the JmjC domain [74], which is a potential functional domain of KDM3 [10]. Among these compounds, JMJD1C Jumonji domain inhibitor 4 (JDI-4), JDI-12, and JDI-16 were identified via surface plasmon resonance analysis as potential KDM3B and KDM3C JmjC domain-binding molecules (Figure 3B). In vitro demethylation assays revealed that JDI-4 reverses H3K9me1 demethylation catalyzed by recombinant KDM3B, which is 57% similar to the JmjC domain of KDM3C. In vivo demethylation assays illustrated that JDI-4 and JDI-12 globally increase H3K9me1 levels. However, Xu et al. did not test the effect of JDIs on the inhibitory activity of other KDMs, including KDM3A. To draw conclusions regarding the biological activity of new inhibitors, it needs to be confirmed that their observed cellular effects are attributable to the inhibition of each KDM3 enzyme rather than non-specific effects or off-target activity, thus whether JDIs can function as novel KDM3-selective inhibitors require further examination.

## 4. Conclusions

Since the discovery of the first histone demethylase in 2004, 30 different demethylases have been identified and investigated [75]. Among them, the KDM3 subfamily of histone demethylases erases specific types of methylation on H3K9, allowing gene transcription. Although their exact physiological roles are not yet fully understood, the KDM3 family members have gained attention because of their essential roles in embryonic development, reproduction, stemness, differentiation, metabolism, and oncogenesis (Figure 2). Among them, KDM3A is especially known to promote cancer cell proliferation, survival, and migration as well as tumor metastasis; it is associated with poor prognosis in various cancer types. Thus, KDM3A is a compelling drug target because of its regulatory roles in chromatin organization and relevance to diseases including cancer.

The latest research in epigenetics and epigenomics uncovered that epigenetic dysregulation is one of the essential causes of tumorigenesis. DNA methylation inhibitors have already been used for cancer treatment [76,77]. In addition to DNA methylation, interest in abnormal histone methylation and its implication in cancer has been growing. Given the number of links of KDM3A to tumorigenesis and metastasis, KDM3A stands as an emerging promising therapeutic target in cancer treatment. Indeed, the structure of some JmjC catalytic domains reveals that they are druggable because they are suitable to embrace small-molecule inhibitors [6,74,78]. Pan-KDM inhibitors have been invented through rational drug design and have been found in compound screens (e.g., JIB-04) [37]. Despite the increasing number of KDM inhibitors in development, only a few drugs have produced satisfactory results and exhibited sufficient specificity for consideration as principal structures for drug development. Consequently, no currently reported compounds are selective for KDM3 subfamily members or their isoforms. Several reasons challenge the development of KDM3-selective inhibitors.

First, most, if not all, KDM3 crystal structures are undetermined. The lack of a critical residue in the Zn-finger domain that specifically recognizes histone substrates at KDM3A makes it more difficult to develop specific inhibitors for KDM3A [10,74]. In contrast to the paucity of structural information regarding KDM3, structural information on KDM4A [6,78], KDM4D [79], KDM5A [80], KDM5B [81], KDM6A [82], and KDM8 [83] has been reported and selective inhibitors based on their structures have been developed [7,74,80,81,82]. Thus, the structure of KDM3 proteins needs to be elucidated to better understand its molecular mechanisms and facilitate the development of potent, and selective KDM3 inhibitors.

Second, most inhibitors are cofactors or substrate mimics with poorly understood mechanisms of action for specific KDM3s. Additionally, other inhibitors identified to date target the broad catalytic machinery of KDM and, therefore, have low specificity for KDM3. Thus, more research is needed to understand the biochemical selectivity of KDM3 to develop specific inhibitors. It is currently known that KDM3A homodimerizes through an association of its catalytic domains and demethylates H3K9 via a two-step process. The two active sites of the homodimer are pivotal for its enzymatic activity [14]. This catalytic mechanism is unlike that of other H3K9 demethylases such as KDM7B/PHF8 and KIAA1718 [84]. These findings hint at the growing possibility of developing small molecules that specifically inhibit KDM3A. Third, no conclusive data have validated the efficacy of KDM inhibitors against specific cancer types. These issues have prevented KDM inhibitors from progressing beyond basic research to clinical research and medical applications. Consequently, further studies are essential to discover promising inhibitors that can selectively inhibit specific KDMs including KDM3A in the vision of treating diseases not limited to cancers.

## Figures and Tables

**Table 1:** Number of published research papers on cancer and KDM3.

KDM3	Histone Substrate	Prostate	Breast	Colon	Lung	Liver	Kidney	Bladder	Head and Neck	Brain	Stomach	Pancreas	Ovary	Cervix	Ewing Sarcoma	Blood	Others	Sum
KDM3A (^1^JMJD1A)	H3K9me1/2	8	8	7	3	4	2	3	2	1	1	1	1	1	2	^4^MM 2	4	50
KDM3B (^2^JMJD1B)	H3K9me1/2	2	1	1	1	1	1	0	0	0	0	0	0	0	0	^5^ALL 2 ^6^AML 3	0	12
KDM3C (^3^JMJD1C)	^7^N/A	2	0	1	0	0	0	0	EC 2	0	1	0	0	0	0	AML 5	1	12
KDM3D (Hairless)	H3K9me1/2	0	0	0	0	0	0	0	0	1	0	0	0	0	0	0	0	1

^1^ JMJD1A: Jumonji domain-containing 1A; ^2^ JMJD1B: Jumonji domain-containing 1B; ^3^ JMJD1C: Jumonji domain-containing 1C; ^4^ MM: multiple myeloma; ^5^ ALL: acute lymphoblastic leukemia; ^6^AML: acute myeloid leukemia; ^7^ N/A: not applicable
